# An Activin A/BMP2 chimera, AB215, blocks estrogen signaling via induction of ID proteins in breast cancer cells

**DOI:** 10.1186/1471-2407-14-549

**Published:** 2014-07-29

**Authors:** Jae Woo Jung, Sun Young Shim, Dong Kun Lee, Witek Kwiatkowski, Senyon Choe

**Affiliations:** Joint Center for Biosciences, Songdo Global University Campus, 187 Songdo-dong, Incheon, 406-840 Yeonsu-gu Korea; Salk Institute for Biological Studies, 10010 N. Torrey Pines RD, La Jolla, CA 92037 USA; Seoul National University, Interdisciplinary Graduate Program in Genetic Engineering, 1 Gwanak-ro, Seoul, Gwanak-gu Korea

**Keywords:** Estrogen receptor-positive breast cancer, Transforming growth factor-β, Bone morphogenetic protein, Tamoxifen alternative, Inhibitor of DNA binding proteins, Bio-better, AB215

## Abstract

**Background:**

One in eight women will be affected by breast cancer in her lifetime. Approximately 75% of breast cancers express estrogen receptor alpha (ERα) and/or progesterone receptor and these receptors are markers for tumor dependence on estrogen. Anti-estrogenic drugs such as tamoxifen are commonly used to block estrogen-mediated signaling in breast cancer. However, many patients either do not respond to these therapies (de novo resistance) or develop resistance to them following prolonged treatment (acquired resistance). Therefore, it is imperative to continue efforts aimed at developing new efficient and safe methods of targeting ER activity in breast cancer.

**Methods:**

AB215 is a chimeric ligand assembled from sections of Activin A and BMP2. BMP2’s and AB215’s inhibition of breast cancer cells growth was investigated. In vitro luciferase and MTT proliferation assays together with western blot, RT_PCR, and mRNA knockdown methods were used to determine the mechanism of inhibition of estrogen positive breast cancer cells growth by BMP2 and AB215. Additionally in vivo xenograft tumor model was used to investigate anticancer properties of AB215.

**Results:**

Here we report that AB215, a chimeric ligand assembled from sections of Activin A and BMP2 with BMP2-like signaling, possesses stronger anti-proliferative effects on ERα positive breast cancer cells than BMP2. We further show that AB215 inhibits estrogen signaling by inducing expression of inhibitor of DNA binding proteins (IDs). Specifically, we demonstrate that knockdown of ID proteins attenuates the anti-estrogen effects of AB215. Remarkably, we find that AB215 is more effective than tamoxifen in suppressing tumor growth in a xenograft model.

**Conclusion:**

This study shows that IDs have profound role to inhibit estrogen signaling in ERα positive breast cancer cells, and that engineered TGF-beta ligands may have high therapeutic value.

**Electronic supplementary material:**

The online version of this article (doi:10.1186/1471-2407-14-549) contains supplementary material, which is available to authorized users.

## Background

Breast cancer is one of the leading causes of death for women worldwide, particularly in developed countries. During the early stage of breast cancer progression, estrogen plays a critical role by enhancing the tumor cell proliferation [1–4]. Estrogen’s pro-oncogenic effect is mediated via nuclear estrogen receptors (ER), ERα and ERβ, by forming steroid/receptor complexes, which in turn interact with DNA at estrogen response elements (EREs) in promoter regions of various genes [5, 6]. This binding of steroid/receptor complex at EREs, requires co-activators including nuclear receptor co-activator 1 (NCOA1), NCOA2, NCOA3 and aryl hydrocarbon receptor nuclear translocator (ARNT), which are all members of basic Helix-Loop-Helix (bHLH) family. Moreover, it was reported that over-expression of NCOAs in breast cancer cells significantly increased their survival [7].

Tamoxifen is an ER antagonist that is currently a major drug used in treatment of ERα-positive (ERα^+^)/pre-menopausal breast cancer patients. Tamoxifen is a competitive antagonist that predominantly blocks the binding of estrogen, 17-β-Estradiol (E2), to ERs. Tamoxifen treatment causes breast cancer cells to remain at the G0 and G1 phase of the cell cycle. Moreover, the ER/tamoxifen complex recruits co-repressors (e.g. Nuclear receptor co-repressor 1 and 2), which in turn stop the genes from being turned on by E2 [8]. However, after prolonged tamoxifen usage, as many as ~30% of breast cancer patients who initially responded to tamoxifen develop resistance to this drug [9, 10]. The mechanism of tamoxifen resistance remains largely unclear and effective alternatives have yet to be discovered.

In addition to estrogen, growth factors including many Transforming Growth Factor-beta (TGF-β) superfamily ligands are also key regulators of ER^+^ breast tumor growth. Bone morphogenetic protein 2 (BMP2) is a TGF-β superfamily member that possesses high affinity for BMP type I receptors (e.g. Activin receptor like kinase 3 [ALK3]) [11, 12] and utilizes the SMAD1/5/8 signaling pathway to induce osteogenesis [13] and chondrogenesis [14]. BMP2 is also reported to suppress the proliferation of MCF7 breast cancer cells by regulating the retinoblastoma [15] and the phosphatase and tensin homolog proteins [16]. However, in contrast to this anti-oncogenic effect, BMP2 has also been reported as a pro-oncogene in breast cancer by promoting cancer cell invasion [17], increasing hormone-independent cancer growth [18], and angiogenesis *in vitro* [19]. Interestingly, it has been reported that E2 treatment mitigated BMP2-induced gene transcription as well as osteoblast differentiation in 2T3 and C2C12 cell lines [20]. Moreover, a BMP2-responsive reporter assay in breast cancer cells displayed a 50% decrease in BMP2 signaling when treated with E2 [21].

Because BMP2 suppresses estrogen-triggered breast cancer cell proliferation, we tested the anti-estrogenic effects of AB215, a chimeric ligand composed of approximately one third Activin A sequence and two thirds BMP2 sequence that possesses enhanced BMP2-like activity. We show that AB215 has stronger anti-estrogenic and anti-proliferative effects on breast cancer cells than BMP2. We further demonstrate that AB215 represses the proliferation of breast cancer cells by inhibiting E2/ERα-mediated signaling via a novel mechanism involving induction of ID proteins. Significantly, we demonstrate that AB215 suppresses ERα^+^ tumor growth and tumor cell proliferation more effectively than tamoxifen in a xenograft model *in vivo*.

## Methods

### Protein preparation

AB215 was prepared as previously described [22]. In brief, Activin A/BMP2 chimeras (AB2 library) have been engineered as a mix of six sequence segments originating from two parental molecules, Activin A and BMP2. AB215 is one such member of AB2 chimera library, which consists of two sequence segments from Activin A and four sequence segments from BMP2 in the order of BABBBA, where A and B denote corresponding segments of Activin A and BMP2, respectively. AB215 was expressed in *Escherichia coli* and chemically refolded [22]. After the purification steps of heparin affinity and C4 reverse phase chromatography, the refolded protein was lyophilized for storage. BMP2 was purchased from joint Protein Central (http://jointproteincentral.com). Prior to use, the lyophilized proteins were reconstituted in 1 mM hydrochloric acid (HCl) in small volume before diluting by at least a factor of 100 in a relevant final buffer or media including phosphate buffered saline (PBS).

### Cell culture

T47D and MCF7 cell lines were purchased from American Type Culture Collection (VA, USA) and SK-BR-3 cell lines from Korean Cell Line Bank (Seoul, Korea). Cells were grown at 37°C humidified atmosphere of 5% CO_2_ in RPMI-1640 medium (Invitrogen, NY, USA) supplemented with 10% fetal bovine serum (FBS) (Invitrogen). All assays were performed in RPMI-1640 without phenol red and supplemented with heat-inactivated and charcoal-stripped FBS (PAA Labs, Pasching, Austria), unless stated otherwise.

### MTT proliferation assay

Cells were plated on a 96-well plate (BD, NJ, USA) at 4×10^3^ cells/well with 2 ~ 5% heat-inactivated and charcoal-stripped FBS. After 24 hours, cells were treated with BMP2, or AB215, with or without 10nM E2 (Sigma) in ethanol. The final concentration of ethanol in all the condition was 0.001% (v/v). After desired period of treatment, 3-(4,5-dimethylthiazol-2-yl)-2,5-diphenyltetrazolium bromide (MTT) reagent (5 mg/ml in PBS, Sigma) was added and incubated at 37°C until purple precipitation was visible. MTT crystal was dissolved in 4 mM HCl, 0.1% NP-40 in isopropanol for 15 minutes and absorbance was measured at 590 nm and baseline corrected at 700 nm.

### Luciferase assay

Cells were plated on a 96-well plate (BD) in Opti-MEM low serum medium (Invitrogen) at 2 × 10^4^ cells/well and reverse co-transfected with ID1-Del2-Luc and β-Galactosidase (β-Gal) using FugeneHD (Roche). After 18 hours of transfection, cells were treated with BMP2 or AB215 with or without 10nM E2. After 24 hours of treatment, cells were lysed using Luciferase lysis buffer (Promega) and their luminescence was measured with plate luminometer (Berthhold, Bad Wildbad, Germany). Transfection variations were normalized by β-gal.

### Western blot

Cells were plated on a 6 or 12-well plate (BD) at 2 × 10^5^ or 1 × 10^5^ cells/well supplemented with 5% heat-inactivated and charcoal-stripped FBS. Cells were treated with 10nM E2, BMP2 or AB215, and exposed for 48 hours. Cells were lysed with cell lysis buffer (Cell Signaling, MA, USA) containing 1 mM PMSF and phosphatase inhibitor cocktail (Roche). Cell lysate’s total protein amount was quantified using Bradford assay. Proteins were separated on SDS-polyacrylamide gels transferred to nitrocellulose (GE healthcare, NJ, USA) or PVDF (Biorad, CA, USA) membrane and analyzed according to the manufacturer’s instruction. Trefoil factor 1 (TFF1) antibody was purchased from Santa Cruz Biotechnology (CA, USA), phosphorylated Extracellular signal-regulated kinases1/2 (ERK1/2), ERK1/2 from Cell Signaling Technology (MA, USA) and β-actin from Sigma.

### Real-time PCR

Cells were plated on a 12-well plate (BD) at 1×10^5^ cells/well supplemented with 5% heat-inactivated and charcoal-stripped FBS. After 16 ~ 24 hours, cells were treated with or without 10nM E2 along with BMP2 or AB215. After 2 ~ 48 hours of treatment, RNA was extracted with TRIsure (Bioline, London, UK) according to the manufacturer’s instruction. cDNA Synthesis was performed using ReverTra Ace qPCR RT Master Mix with gDNA remover (Toyobo, Japan) according to the manufacturer’s instruction. Analysis of mRNA expression was determined with quantitative real-time polymerase chain reaction (qRT-PCR) using Thunderbird SYBR qPCR mix (Toyobo), and 10 pM primers according to the manufacturer’s instruction. The sequences of primers are listed in Table 1. Abundance of mRNA in each sample was determined by the differences between the cycle threshold (Cτ) values for each genes and β-actin, ΔCτ. Relative ratios of mRNA expression levels were defined as 2^−ΔΔCτ^, where ΔΔCτ = ΔCτ_sample_-ΔCτ_control_, which reflect changes of mRNA expression levels from treated cells compared to those from untreated cells. All experiments were performed at least 3 times with triplicate samples.

**Table 1:** **List of RT-PCR primers**

mRNA	Sequence
Human β-actin	FWD-5′-GGATCAGCAAGCAGGAGTATG
	REV-5′-AGAAAGGGTGTAACGCAACTAA
Human TFF1	FWD-5′-CCCCTGGTGCTTCTATCCTAAT
	REV-5′-CAGATCCCTGCAGAAGTGTCTA
Human Bcl2	FWD-5′-ATGTGTGTGGAGAGCGTCAACC
	REV-5′-TGAGCAGAGTCTTCAGAGACAGCC
Human c-myc	FWD-5′-CTGAGGAGGAACAAGAAGATGAG
	REV-5′-TGTGAGGAGGTTTGCTGTG
Human VEGF	FWD-5′-CTACCTCCACCATGCCAAGT
	REV-5′-GCAGTAGCTGCGCTGATAGA
Human Cathepsin D	FWD-5′GACCAGAACATCTTCTCCTTCTAC
	REV-5′GGACAGAGAACCCTTGTAATACTT
Human ERα	FWD-5′GGCTTCTCTTGGTATGTCTTGT
	REV-5′CTCCCAGATTCTCAGTCCTTTG
Human SMAD1	FWD-5′CTACCCTCACTCTCCCACCA
	REV-5′GCACCAGTGTTTTGGTTCCT
Human SMAD5	FWD-5′CCCAAGGATAAGGCTACTGATTT
	REV-5′TCCCAAAGTGCTGGGATTAC
Human SMAD8	FWD-5′CAAGAAGCAGGTGAAACCAAAG
	REV-5′AGACTGGAACGTGGGAAATG
Human SMAD4	FWD-5′TTGCGTCAGTGTCATCGACAG
	REV-5′CCAGCCTTTCACAAAACTCATCC
Human BMPRIa	FWD-5′CCAGTCACAAAGTTCTGGTAGT
	REV-5′CTTCTCCATATCGGCCTTTACC
Human BMPRIb	FWD-5′GGAACTCTGCTGGAAGGTAAA
	REV-5′CCGTTCTATGTCCTCCAACTTAG
Human BMPRII	FWD-5′CAAGCAAAGACTGGTGACTTTATC
	REV-5′GATAGCAGCCCTTCCTTCATAG
Human ID1	FWD-5′-TTACGTGCTCTGTGGGTCTC
	REV-5′-CCCCCTAAAGTCTCTGGTGA
Human ID2	FWD- 5′-ATGAAAGCCTTCAGTCCCGT
	REV- 5′-TTCCATCTTGCTCACCTTCTT
Human ID3	FWD- 5′-TCATCTCCAACGACAAAAGG
	REV- 5′-ACCAGGTTTAGTCTCCAGGAA
Human ID4	FWD- 5′-TGAACAAGCAGGGCGACA
	REV- 5′-CGTGCAAAGAAAGAATGAAAG

### mRNA knockdown

Genes of interest were knocked down using small interference RNA (siRNA) transfection. siRNA duplex was purchased/synthesized from Bioneer Inc (Korea). Cells were reverse transfected with siRNA duplex complexed with Lipofectamine RNAiMAX (Invitrogen) reagent in serum free RPMI1640 media without phenol red (Invitrogen) as specified by manufacturer’s instruction. Briefly, 15 pmol siRNA duplex was diluted in 200 ul serum free RPMI1640 without phenol red and complexed with Lipofectamine for15 ~ 20 minutes. 1×10^5^ cells in RPMI1640 supplemented with10% heat-inactivated and charcoal-stripped FBS were added to the mixture in each well in a 12 well plate. Cells were treated with ligands after 24 ~ 48 hours of transfection. We tested 1 ~ 3 siRNAs from Bioneer to select the most efficient construct. The following sequences of siRNAs for particular gene knockdowns were used; ID1- FWD-5′-UCGCAUCUUGUGUCGCUGA, REV-5′-UCAGCGACACAAGAUGCGA; ID2- FWD-5′-CUUACUUGGACUGUGAUAU, REV-5′-AUAUCACAGUCCAAGUAAG; ID3- FWD-5′-CUGUAACAAUGCGAUGUAU, REV-5′-AUACAUCGCAUUGUUACAG; ID4- FWD-5′-GUGACAUUUCAUACCAUGU, REV-5′-ACAUGGUAUGAAAUGUCAC. Negative control was transfected with *AccuTarget* Negative control siRNA (Bioneer). Knockdown (KD) efficiency was determined by qRT-PCR.

### *In vivo*tumor xenograft model

Continuous E2-releasing pellets for 90 days (Innovative Research of America, FL, USA) were implanted subcutaneously into 4–6 weeks old KSN/Slc athymic mouse (n = 5) 3 days before xenograft. MCF7 breast cancer cells (5×10^6^ cells) were subcutaneously xenografted in 50 μl RPMI1640 with 50 μl Matrigel Matrix (BD) using 21-gauge needle on the dorsal side. The ligand injection started when tumor was visible (after 17 days). Two doses (0.12 (low) or 0.4 (high) mg/kg of mice) of AB215 and 0.6 mg/kg dose of tamoxifen were subcutaneously injected, three times a week for 10 weeks (10 weeks total - low group- ~ 90 ug, high group- ~ 300 ug injected). After 70 days from injection started, mice were sacrificed, and tumor was surgically removed. Mice were also examined for tumors in other organs and the spleen size was measured to evaluate inflammation. All the *in vivo* experiments were done under the guideline of AAALAC (Association for Assessment and Accreditation of Laboratory Animal Care International). All the procedures were performed at the Lee Gil Ya Cancer and Diabetes Institute and approved by Institutional Animal Care and Use Committee (IACUC No. 2011–0103) at Gachon University in South Korea.

### Immunohistochemistry

Tumor tissues were fixed in formaldehyde, embedded in paraffin, sectioned, deparaffinized/hydrated and processed for antigen retrieval by microwaving 3 times for 5 minutes in 10 mM Tris–HCl/pH9.0 and 1 mM EDTA. The sections were then incubated with Ki67 antibody (Santa Cruz Biotechnology) at 4°C overnight and analyzed using ImmPress peroxidase polymer detection kit (Vector Labs, CA, USA). Harris Hematoxylin (BBC, WA, USA) was used for counter stain by following standard protocol.

### Cell invasion assay

A fluorometric kit for cell invasion assay was purchased from Cell Biolabs (CA, USA). All the procedures followed the manufacturer’s protocol. Briefly, 2 × 10^6^ cells were plated on upper chamber of transmembrane-welled plates in serum-free RPMI 1640 medium with or without ligands. Lower chamber contained 10% serum or 10nM E2. After 18 hours, penetrated cells were analyzed using CyQuant reagent and quantified by a multi-well fluorometer.

### Statistical/graphical analysis

All the numerically quantifiable data have been statistically analyzed and graphically presented using Prism software (Graphpad, CA, USA). Column analysis was performed by one-way ANOVA with Dunnett’s post-hoc test adjustment.

## Results

### AB215 strongly induces ID proteins

**Figure 1:**
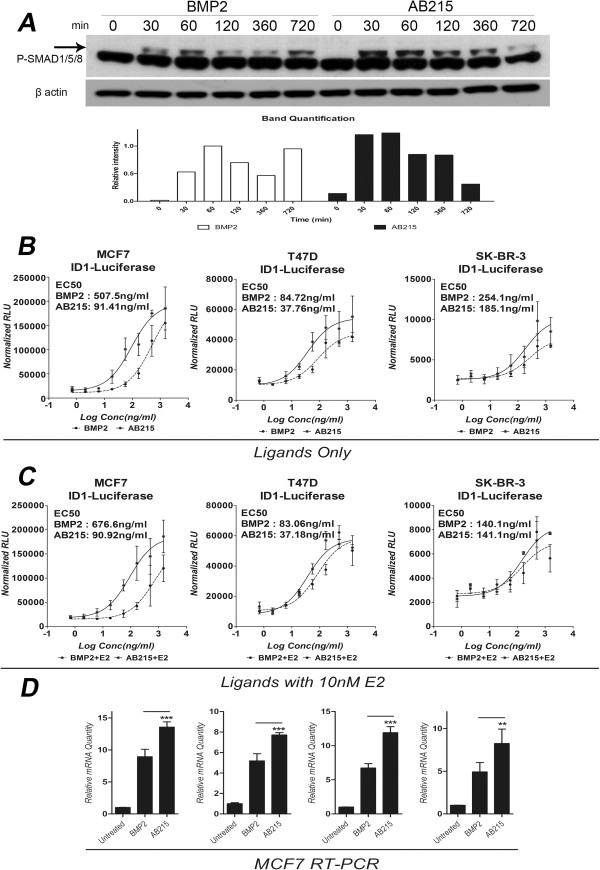
**Enhanced signaling capacity of AB215 compared to BMP2 in breast cancer cells. A)** Time course western blot analysis of MCF7 cells was performed after exposing BMP2 and AB215 for indicated time. The arrow points to the ligand-induced upper band. **B**-**C)** ID1-driven Luciferase reporter assay of BMP2 and AB215 for MCF7, T47D and SK-BR-3 cells in the **B)** absence and **C)** presence of 10nM E2. Cells were reverse transfected with ID1-Luciferase reporter plasmid in triplicate. Transfected cells were exposed to ligands for 18 hours. Relative luciferase value has been normalized with β-gal. The data is shown in means ± SD, (n: number of independent experiments = 3). **D)** mRNA level of IDs in MCF7 cells. BMP2 and AB215 induced mRNA expression level of ID1/2/3/4 has been measured by qRT-PCR in MCF7 cells. Cells were exposed to 500 ng/ml of BMP2 and AB215 for 18 hours. Relative mRNA level was calculated in quadruplicate using ΔΔCτ method with the control and β-actin as a reference. All data are shown in means + SD, n = 3. (* = P ≤ 0.05, ** = P ≤ 0.01, *** = P ≤ 0.001).

We previously reported that AB215 signals via SMAD1/5/8 pathway and possesses enhanced signaling relative to BMP2 in the C2C12 mouse myoblast cell line [22]. Here we also show that, as predicted, AB215 does not signal through SMAD2/3 and, therefore, does not signal in an Activin A-like manner in HEK293T cells (Additional file 1: Figure S1). We further examined the signaling properties of AB215 in human MCF7 breast cancer cells and found that, similar to what was observed in C2C12 cells, AB215 produces prolonged and enhanced SMAD1/5/8 phosphorylation when compared to that induced by BMP2 (Figure 1A). The level of BMP2-induced SMAD1/5/8 phosphorylation in MCF7 cells peaks after 60 minutes and then decreases to basal levels after 3 hours. By contrast, treatment of these cells with AB215 results in maximal SMAD1/5/8 phosphorylation 30 min following stimulation and sustained after 6 hours (Figure 1A). We also used a reporter construct consisting of the phospho-SMAD1/5/8 responsive ID1 promoter upstream of a luciferase gene to compare the effects of BMP2 and AB215 treatment on the human breast cancer cell lines MCF7, T47D and SK-BR-3 in the absence (Figure 1B) or presence (Figure 1C) of E2 treatment. Our results show that AB215 is more potent and has greater efficacy than BMP2 in these cell lines and that E2 does not produce statistically significant effect on ligand-induced ID1 promoter activation of AB215 (Figure 1B and C). In addition, we used qRT-PCR to demonstrate that AB215 induces expression levels of all four ID proteins, ID1, ID2, ID3 and ID4, in MCF7 cells to a greater extent than BMP2 (Figure 1D).

### AB215 inhibits estrogen-induced growth of ERα^+^ cells

**Figure 2:**
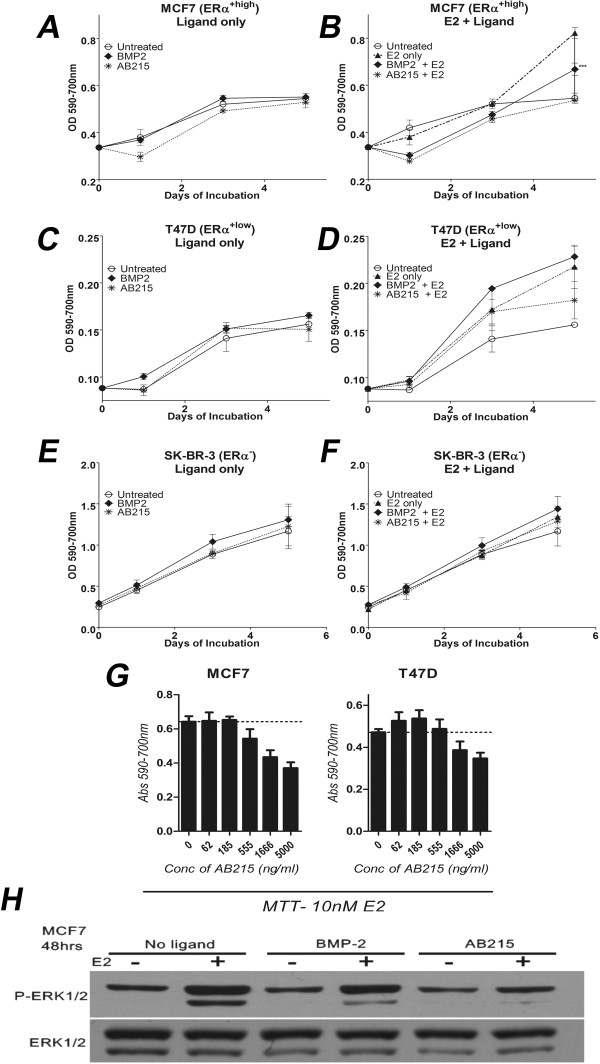
**Anti-proliferative property of AB215 on ERα** ^**+**^ **breast cancer cells. A, B)** ERα^+^: MCF7, **C, D)** T47D and **E, F)** ERα^−^: SK-BR-3, cells were grown in phenol red free RPMI1640 supplemented with 2% heat inactivated charcoal-stripped FBS treated with or without 10nM E2 along with 500 ng/ml BMP2 or AB215 in quintuplicate. Cell proliferation was analyzed by MTT assay (Abs590-700 nm) on 0, 1, 3 and 5 days after the treatment (n = 3). E2 and AB215 did not affect the proliferation of ERα^−^ cells significantly. The results are presented as means ± SD and their significance has been analyzed by one-way ANOVA. **G)** MTT assay of MCF7 and T47D cells at increasing concentration of AB215 in the presence of 10nM E2. Cells were plated and treated as explained in Figure 2a-f. Cells were analyzed 4 days after the treatment. Data are shown in means + SD. **H)** Western blot analysis of E2 induced phosphorylation of ERK1/2. Cells were plated in phenol red free RPMI1640 supplemented with 5% heat inactivated charcoal-stripped FBS treated with or without 10nM E2 along with 500 ng/ml BMP2 or AB215. Cells were harvested and lysed after 48 hours of exposure for western blot analysis. (* = P ≤ 0.05, ** = P ≤ 0.01, *** = P ≤ 0.001).

We investigated the ability of AB215 to inhibit the growth of ERα^+^ MCF7 and T47D as well as ERα-negative (ERα^−^) SK-BR-3 human breast cancer cells. Although MCF7 and T47D cells are both ERα^+^, the expression level of ERα is about 4-fold higher in MCF7 cells than in T47D (Additional file 2: Figure S2a) [23]. We treated cells with AB215 or BMP2 in the presence or absence of E2 and found that AB215 inhibits E2-induced growth of MCF7 (Figure 2A and B) and T47D (Figure 2C and D) cells. MCF7 (ERα^+High^) cells were more sensitive to inhibition than T47D (ERα^+Low^) cells. BMP2 also inhibits MCF7 cell proliferation but to a lesser extent than AB215 and has no statistically relevant effect on the proliferation of T47D cells. On the other hand, neither AB215 nor BMP2 affected proliferation of ERα^−^, SK-BR-3 (Figure 2E and 2F). It is important to note that the anti-proliferative effect of AB215 depends on its concentration in both MCF7 and T47D cells (Figure 2G).

One of the key mechanisms of estrogen-induced proliferation of breast cancer cells and tumor progression is the activation of mitogen activated protein kinase, by promoting phosphorylation of ERK1/2 [24]. Consistent with its ability to block estrogen-induced proliferation, AB215 inhibits estrogen-induced phosphorylation of ERK1/2 in MCF7 cells and does so more strongly than BMP2 (Figure 2H).

### AB215 blocks estrogen-induced ERK signaling by inducing ID proteins

**Figure 3:**
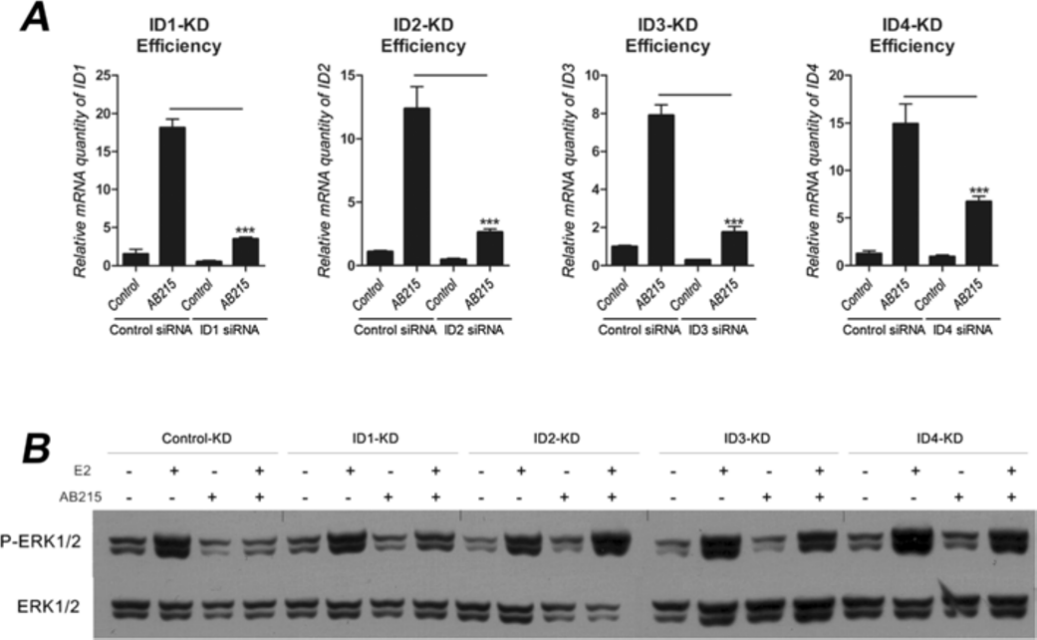
**AB215 induced IDs cooperatively inhibit E2 activating P-ERK1/2.** MCF7 cells were reverse transfected with siRNA in serum free/phenol red free RPMI1640 and plated in phenol red free RPMI1640 supplemented with 10% heat inactivated charcoal-stripped FBS. Control was transfected with non-sense siRNA. After 24 hours of transfection, cells were treated with or without 10nM E2 along with 500 ng/ml BMP2 or AB215. After 48 hours of exposure, RNA was extracted for qRT-PCR analysis or harvested for western blot analysis. **A)** KD efficiency of each IDs using siRNA. mRNA level of each IDs were measured using qRT-PCR to confirm the effectiveness of mRNA knocked down. Data are shown as means + SD in quadruplicates, n = 3. **B)** KD samples are analyzed for western blot to detect phosphorylation of ERK1/2. The figure shown is done in two separate blots (Control, ID1, ID2-KDs in one blot and ID3, ID4-KDs in another blot). (* = P ≤ 0.05, ** = P ≤ 0.01, *** = P ≤ 0.001).

Since AB215 inhibits E2-induced growth of ERα^+^ breast cancer cells and ERK1/2 signaling, we hypothesized that AB215 induction of ID proteins plays a role in this inhibition. ID proteins belong to bHLH family of transcription factors. They possess a HLH domain that allows them to heterodimerize with other bHLH transcription factors, but they lack a DNA binding domain and therefore act as inhibitors of other transcription factors. Hence, we hypothesized ID proteins may inactivate HLH co-activators of E2/ER assembly such as NCOAs and ARNT by forming nonproductive complexes with them and thereby preventing the assembly competent DNA-binding complexes. To test this hypothesis, we transiently knocked down each of the ID mRNAs using siRNA in ERα^high^ MCF7 cells and investigated the resulting effect of AB215 treatment on E2-induced ERK1/2 phosphorylation in these cells. The efficiency of ID-KD was confirmed by comparing the ability of control or ID-specific siRNAs to block AB215-induced ID expression (Figure 3A). Our knockdown studies revealed that all four ID proteins, but especially ID2, ID3 and ID4, play key roles in mediating AB215 inhibition of E2-induced ERK1/2 phosphorylation (Figure 3B). Furthermore, our results suggest that these ID proteins are not redundant, but rather that there is a cooperativity between them in mediating this inhibition process since the inhibitory effect of AB215 is severely diminished by knocking down ID2, ID3 or ID4 separately (Figure 3B).

### AB215 inhibits expression of E2-induced genes

**Figure 4:**
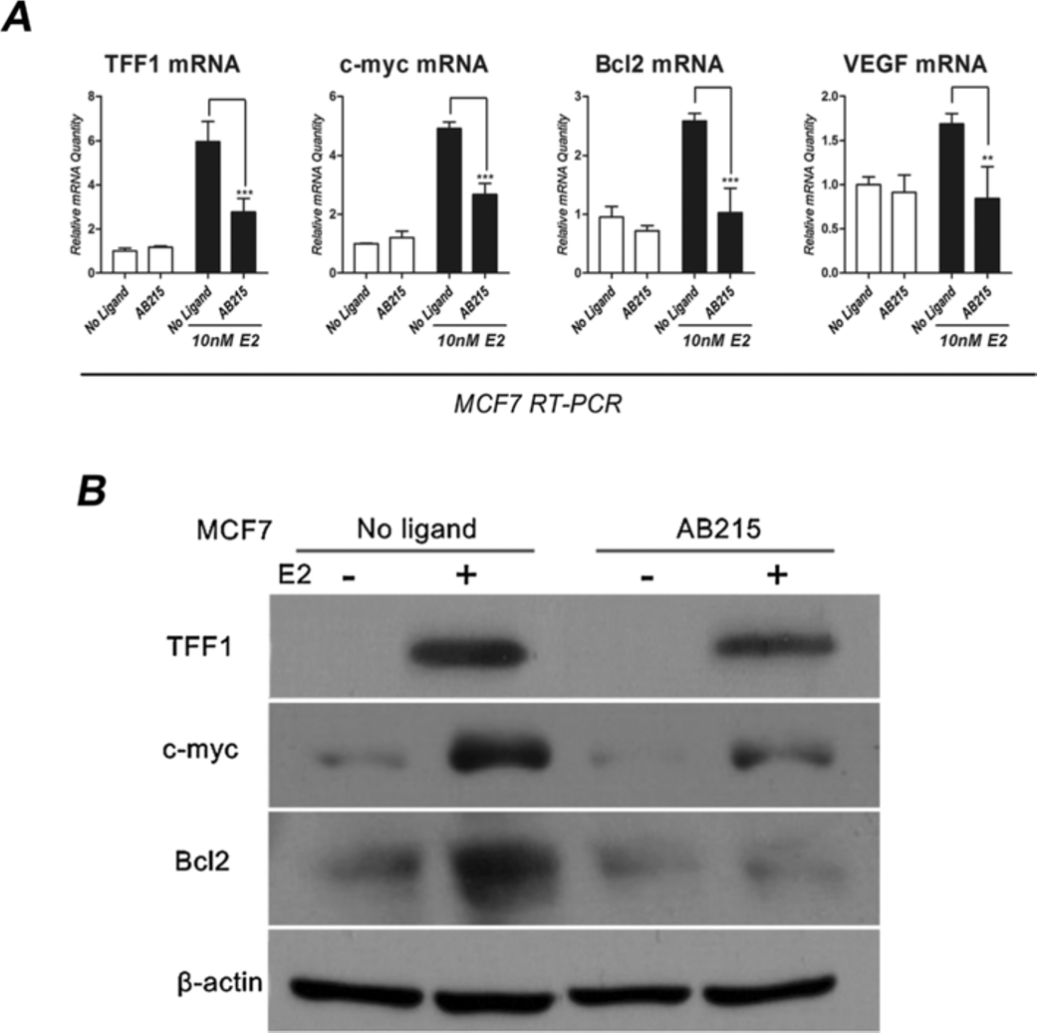
**AB215 inhibits estrogen induced signaling.** To investigate the effect of AB215 on Estrogen signaling, **A)** mRNA levels of TFF1, c-myc, Bcl2 and VEGF and **B)** protein levels of TFF1, c-myc, Bcl2 were analyzed in MCF7 cells. Cell were exposed to the treatment (500 ng/ml AB215 and 10nM E2) for 48 hours (TFF1, Bcl2, and VEGF) and 2 hours (c-myc). qRT-PCR and western blot analysis was performed as described in Figure 3 and experimental conditions are same as in Figure 2i. mRNA levels are presented as means + SD of quadruplicate, n = 3 and their significance has been analyzed by one-way ANOVA (* = P ≤ 0.05, ** = P ≤ 0.01, *** = P ≤ 0.001).

TFF1 is a peptide that is expressed at low levels in normal breast tissue, [25–27] but at high levels in ERα^+^ breast carcinomas [28] in response to E2 [29]. Since TFF1 is strictly controlled by the E2/ERα complex, it provides a good measure of estrogen signaling in breast cancer cells and a preliminary clinical study reported a parallel relationship between the TFF1 high expression levels and the proliferation of breast cancer cells [30]. Oncogenes *Bcl2* [31], *c-myc* [8, 32] and Vascular Endothelial Growth Factor (*VEGF*) [33] are also reported to be a breast cancer-specific estrogen-responsive genes. We investigated the effects of AB215 treatment on the expression of these genes in the absence or presence of estrogen treatment in ERα^high^ MCF7 cells. RT-PCR and western blot analysis shows that E2-induced TFF1, c-myc, Bcl2, and VEGF mRNA (Figure 4A) and TFF1, c-myc, Bcl2 protein (Figure 4B) levels are increased by estrogen treatment and this effect is significantly suppressed by co-administration with AB215.

### AB215 reduces *in vivo*growth of breast cancer cells

**Figure 5:**
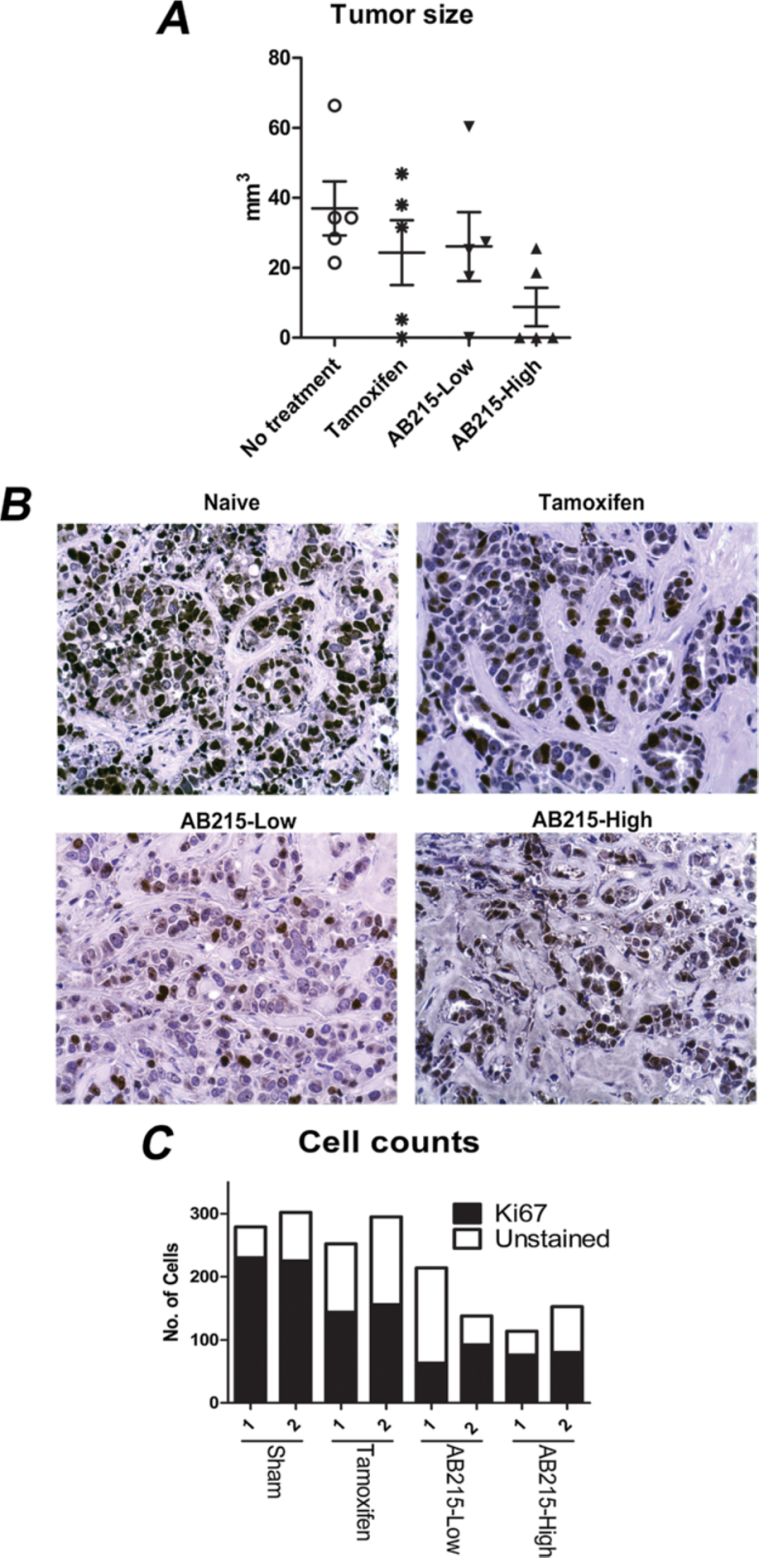
***In vivo*** **anti-proliferation property of AB215** ***.*** Athymic nude mice implanted with continuous releasing E2 pellet were xenografted with MCF7 cells in the dorsal region. Low (0.12 mg/kg · injection) or high (0.4 mg/kg · injection) dose of ligand or 0.6 mg/kg tamoxifen was injected in q2d × 10 (every 2 days for 10 weeks) schedule. **A)** Collected tumors were measured for volume according to π/6 × (length) × (width) × (height) equation (n = 5). Results are presented in means + SD. **B)** Collected tumors were analyzed to determine levels of nuclear proliferation marker Ki67 (final magnification 400×). Each section was counter stained with hematoxylin (blue/purple) to distinguish proliferating and non-proliferating cells. **C)** The quantification of the images from two different tumor sections representing the total number of cells and the number of cells labeled with Ki67.

The anti-proliferative activity of AB215 *in vitro* prompted us to investigate its potential anti-tumor effects *in vivo*. We compared the effects of AB215 with those of tamoxifen, an anti-estrogenic drug widely used to treat ER^+^ breast cancer patients. AB215 and tamoxifen both appeared to reduce the size of tumor xenografts following 3 months of treatment in the presence of an E2 release pellet (Figure 5A).To further compare the effects of AB215 and tamoxifen on tumor progression, we measured the expression patterns and levels of the nuclear proliferation marker Ki67. As shown in Figure 5B, both AB215 and tamoxifen treatments were effective in reducing cancer cell proliferation. However, both the high and low dose AB215 treatments resulted in noticeably lower cancer cell density than the untreated and the tamoxifen-treated tumors (Figure 5C).

## Discussion

We constructed the AB2 library of segmental chimeras between Activin A and BMP2 [22] in order to create novel ligands with unique structural and functional properties and the potential to fulfill medical needs. The present study provides evidence that one of these, AB215 (BABBBA), can inhibit estrogen signaling and the growth of estrogen-fueled ER^+^ breast tumors. From the three-dimensional structure of the ternary complex of BMP2, Activin receptor Type II (ActRII)-Extracellular domain (ECD), and ALK3-ECD [34] it can be inferred that most of the type II receptor binding site of AB215 consists of Activin A sequence while almost all of its type I receptor binding site is derived from BMP2. Since both BMP2 and Activin A utilize the type II receptors ActRII and ActRIIb, we hypothesized that a chimeric ligand that possesses the type I receptor specificity of BMP2 together with the high affinity type II receptor binding properties of Activin A may have enhanced BMP2-like properties. Indeed, AB215 signals via the SMAD1/5/8 pathway but not the SMAD2/3 pathway and has increased potency relative to BMP2.

BMP2 can inhibit the progression of many different types of cancers but its role is also bi-directional since it is also implicated in tumor progression and angiogenesis in some cancers. Since BMP2 inhibits proliferation of ERα^+^ breast cancer cells, we hypothesized that the increased BMP2-like signaling activity of AB215 may augment AB215’s potency in anti-proliferation of ERα^+^ breast cancer cells. In the present study, we established that AB215 indeed inhibits E2-induced proliferation of ERα^+^ breast cancer cells to a greater extent than BMP2. Furthermore, like BMP2, AB215 has no proliferative effect on ERα^−^ cells indicating that both ligands exert their anti-proliferative effects through effects on E2 signaling. Results led us to conclude that the anti-proliferative effects of AB215 are not only dependent on the ERα status, but also on the level of ERα expression since it had less of an effect on the proliferation and E2-induced gene expression in T47D cells which express ERα at lower levels than in MCF7 cells (Additional file 2: Figure S2a-e). The fact that T47D cells were less susceptible to AB215’s anti-proliferative effects than MCF7 cells (Figure 2B and 2D) strongly indicates that these effects are at least partially exerted via E2/ERα signaling.

E2-induced phosphorylation of ERK is thought to play essential role in mediating increases in cellular proliferation. Although the mechanism of E2-induced ERK phosphorylation remains unclear, epidermal growth factor receptor, protein kinase Cδ and HER-2/neu have each been shown to be involved [24]. Here, we show that AB215 can inhibit E2-induced ERK phosphorylation and E2/ERα-induced gene expression. Consistent with our working hypothesis that AB215 blocks E2 signaling by inhibiting E2/ERα complex binding to EREs of various genes, we found that ID proteins are significantly up regulated downstream of AB215 signaling, and thus play a critical role in mediating inhibition of E2-induced ERK phosphorylation. We propose that ID proteins may interfere with the binding of E2/ERα to EREs by sequestering the E2/ER co-activator proteins such as NCOA and ARNT in nonproductive complexes. Intriguingly, our results also demonstrate that ID proteins act in a non-redundant and highly cooperative manner. Future studies will elucidate the precise mechanism through which ID proteins block E2-induced gene regulation.

Our *in vivo* studies demonstrate that the anti-tumorigenic effects of AB215 are similar to those of tamoxifen, not only in reducing tumor size, but also in improving tumor grade according to Ki67 expression level. It is important to note that prolonged injections of high concentration of AB215 had no apparent toxicity to mice and none of these mice developed abnormalities such as weight loss (Additional file 3: Figure S3), inflammation or tumorigenesis. Moreover, *in vitro* cell invasion assays of AB215-treated MCF7 cells did not show development of characteristic metastatic properties. (Additional file 4: Figure S4).

## Conclusions

We show that the Activin A/BMP2 chimera AB215 strongly induces ID proteins and thereby interferes with the pro-proliferative and gene expression effects of E2/ERα signaling. Furthermore, our results suggest that this enhanced BMP2-like molecule is at least as efficient as tamoxifen in reducing the size of tumors resulting from breast cancer xenografts highlighting its potential effectiveness for the treatment of breast tumors, especially those resistant to tamoxifen. This discovery puts AB215 in a prime position as a novel endocrine therapeutic biologic and opens a new inroad to study the complex mechanisms regulating estrogen-driven cancer cell proliferation.

## Electronic supplementary material

Additional file 1: Figure S1: Activin A signaling ability of AB215. To analyze the Activin A signaling (SMAD2/3) capacity of AB215, Activin responsive element (ARE) driven luciferase assay was performed in HEK293T cells. Cells were reverse co-transfected with ARE-Luciferase and β-galactosidase plasmid. Transfected cells were treated with Vehicle, Activin A (100 ng/ml), BMP2 (500 ng/ml) and AB215 (500 ng/ml) for 24 hours and lysed for Luciferase activity. The experiments were done in triplicates and transfection differences were normalized using β-gal. The data are shown as means + SD. (PDF 8 MB)

Additional file 2: Figure S2: Basal mRNA expression level of ERα^high^MCF7 and ERα^low^T47D. Cells were plated in phenol red free RPMI1640 supplemented with 10% heat inactivated charcoal-stripped FBS and harvested after 48 hrs for RNA extraction. cDNA was synthesized and RT-PCR was performed to determine basal expression level of a) Estrogen signaling components: ERα, TFF1, Bcl2, c-myc and Cathepsin D, b) AB215/BMP2 signaling components: SMAD1, SMAD5, SMAD8 and SMAD4, c) AB215/BMP2 receptors: BMPRIa (ALK3), BMPRIb (ALK6) and BMPRII, and d) Inhibitor of DNA binding proteins (ID): ID1, ID2, ID3 and ID4. (PDF 11 MB)

Additional file 3: Figure S3: Lethality test of AB215 in CD-1/ICR mice. To verify the lethality and ectopic organ bone formation of the AB215 in the body, AB215 was injected intraperitoneally injected three times a week for 98 day at two concentrations (15 ug/kg and 600 ug/kg, n = 5). The body weight was measured three times a week and is shown as mean + SD. (PDF 11 MB)

Additional file 4: Figure S4: AB215 (and BMP2) *in vitro* basal membrane invasion test. MCF7 and T47D cells were treated as defined and analyzed for basement membrane matrix invasion ability as described in the methods section. As an attractant, 10% FBS (a and b) and 10nM E2(c and d) were used. Results are presented as means + SD. (PDF 10 MB)

## Abbreviations

ER: Estrogen receptor
ERE: Estrogen responsive element
NCOA: Nuclear co-activator
ARNT: Aryl hydrocarbon receptor nuclear translocator
bHLH: Basic Helix-Loop-Helix
E2: 17-β-Estradiol
TGF-β: Transforming growth factor-beta
BMP: Bone morphogenetic protein
ALK: Activin receptor like kinase
ID: Inhibitor of DNA binding protein
HCl: Hydrochloric acid
PBS: Phosphate buffered saline
FBS: Fetal bovine serum
MTT: 3-(4,5-dimethylthiazol-2-yl)-2,5-diphenyltetrazolium bromide
β-Gal: β-Galactosidase
TFF1: Trefoil factor 1
ERK1/2: Extracellular signal-regulated kinases1/2
Cτ: Cycle threshold
siRNA: Small interference RNA
KD: Knockdown
VEGF: Vascular Endothelial Growth Factor
ActRII: Activin receptor Type II
ECD: Extracellular domain.
