# Supplementary figures and images for: An Activin A/BMP2 chimera, AB215, blocks estrogen signaling via induction of ID proteins in breast cancer cells
Source: BMC Cancer. 2014 Jul 29;14:549. doi: 10.1186/1471-2407-14-549 (PMC4122783; doi:10.1186/1471-2407-14-549)

# Supplemental Figure 1

**a**

**HEK293T-ARE (SMAD2/3)**

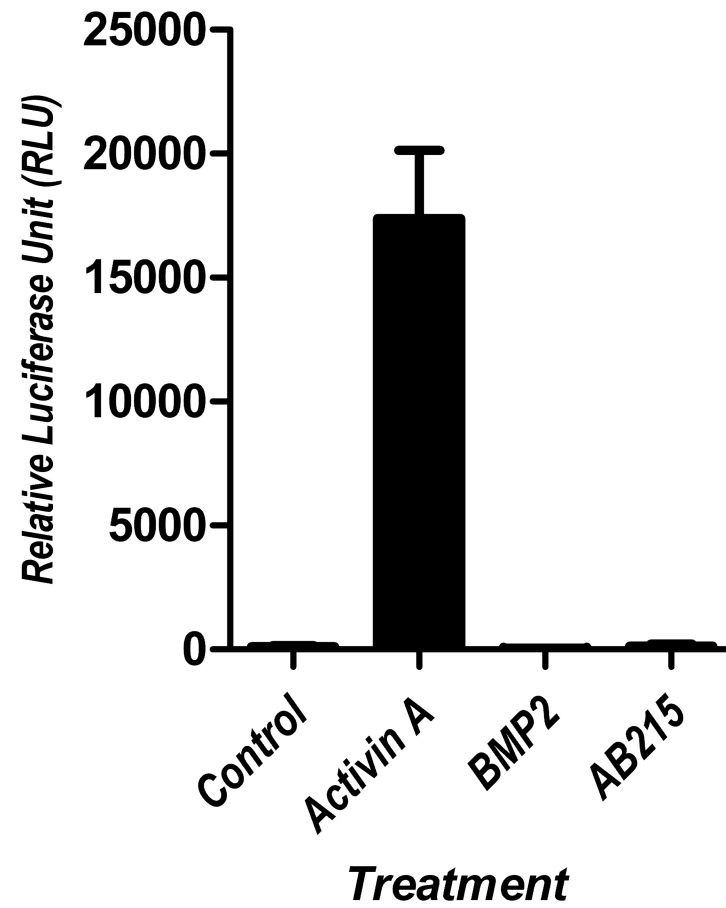

Supplement: Supplementary file 1 — Additional file 1: Figure S1: Activin A signaling ability of AB215. To analyze the Activin A signaling (SMAD2/3) capacity of AB215, Activin responsive element (ARE) driven luciferase assay was performed in HEK293T cells. Cells were reverse co-transfected with ARE-Luciferase and β-galactosidase plasmid. Transfected cells were treated with Vehicle, Activin A (100 ng/ml), BMP2 (500 ng/ml) and AB215 (500 ng/ml) for 24 hours and lysed for Luciferase activity. The experiments were done in triplicates and transfection differences were normalized using β-gal. The data are shown as means + SD. (PDF 8 MB) [file 12885_2014_4723_MOESM1_ESM.pdf]

# Supplemental Figure 2

**a**

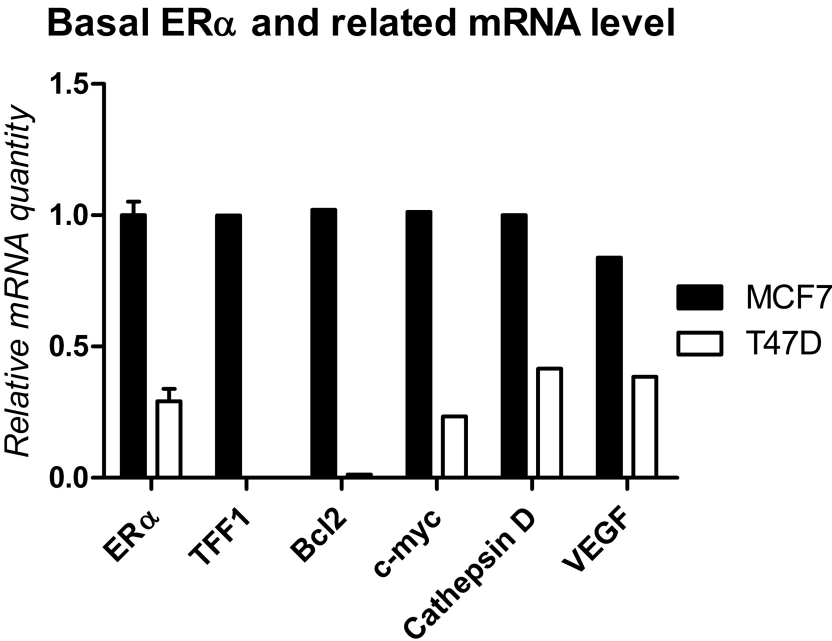

**b**

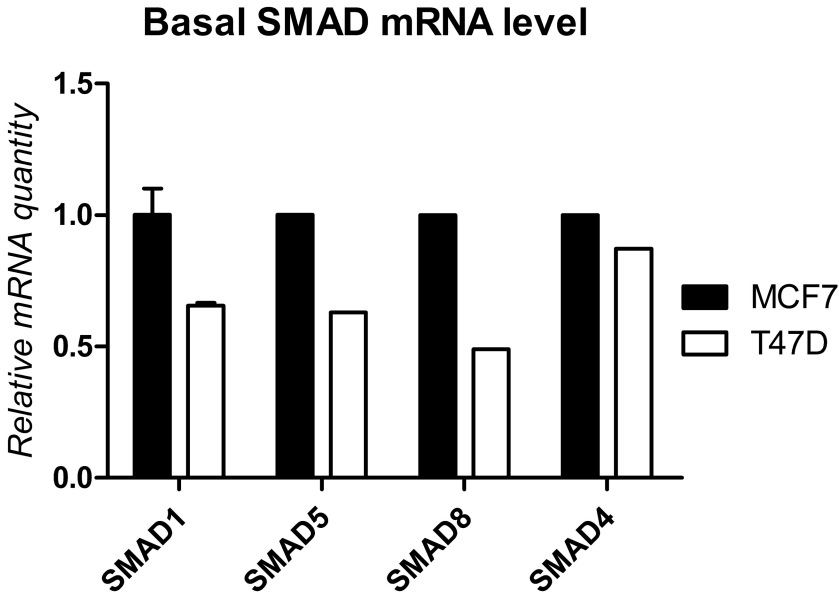

**c**

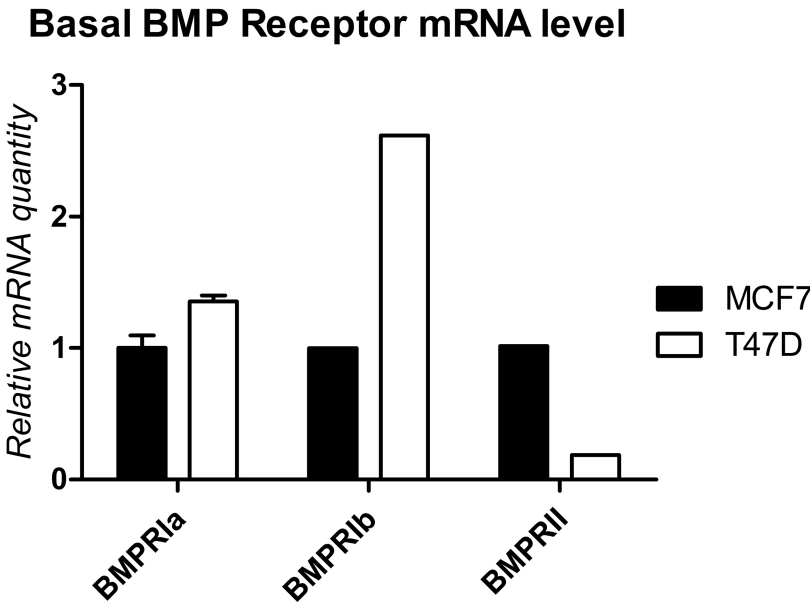

**d**

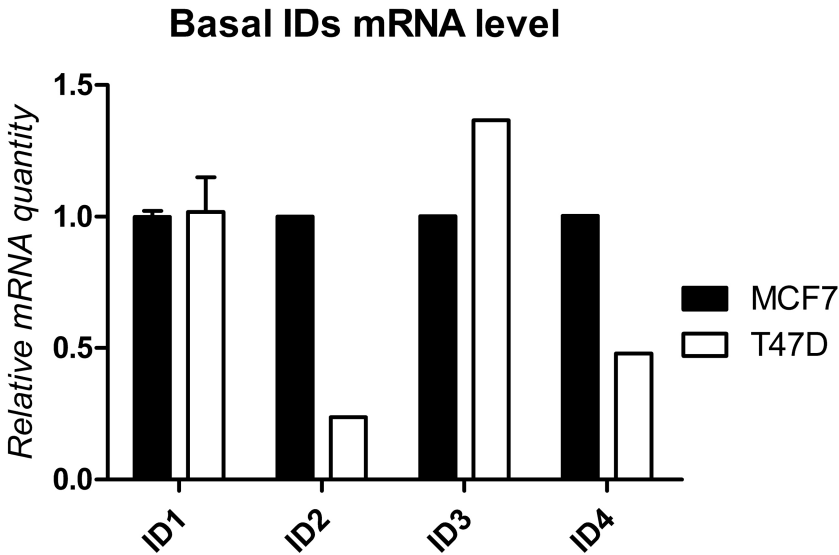

Supplement: Supplementary file 2 — Additional file 2: Figure S2: Basal mRNA expression level of ERαhighMCF7 and ERαlowT47D. Cells were plated in phenol red free RPMI1640 supplemented with 10% heat inactivated charcoal-stripped FBS and harvested after 48 hrs for RNA extraction. cDNA was synthesized and RT-PCR was performed to determine basal expression level of a) Estrogen signaling components: ERα, TFF1, Bcl2, c-myc and Cathepsin D, b) AB215/BMP2 signaling components: SMAD1, SMAD5, SMAD8 and SMAD4, c) AB215/BMP2 receptors: BMPRIa (ALK3), BMPRIb (ALK6) and BMPRII, and d) Inhibitor of DNA binding proteins (ID): ID1, ID2, ID3 and ID4. (PDF 11 MB) [file 12885_2014_4723_MOESM2_ESM.pdf]

# Supplemental Figure 3

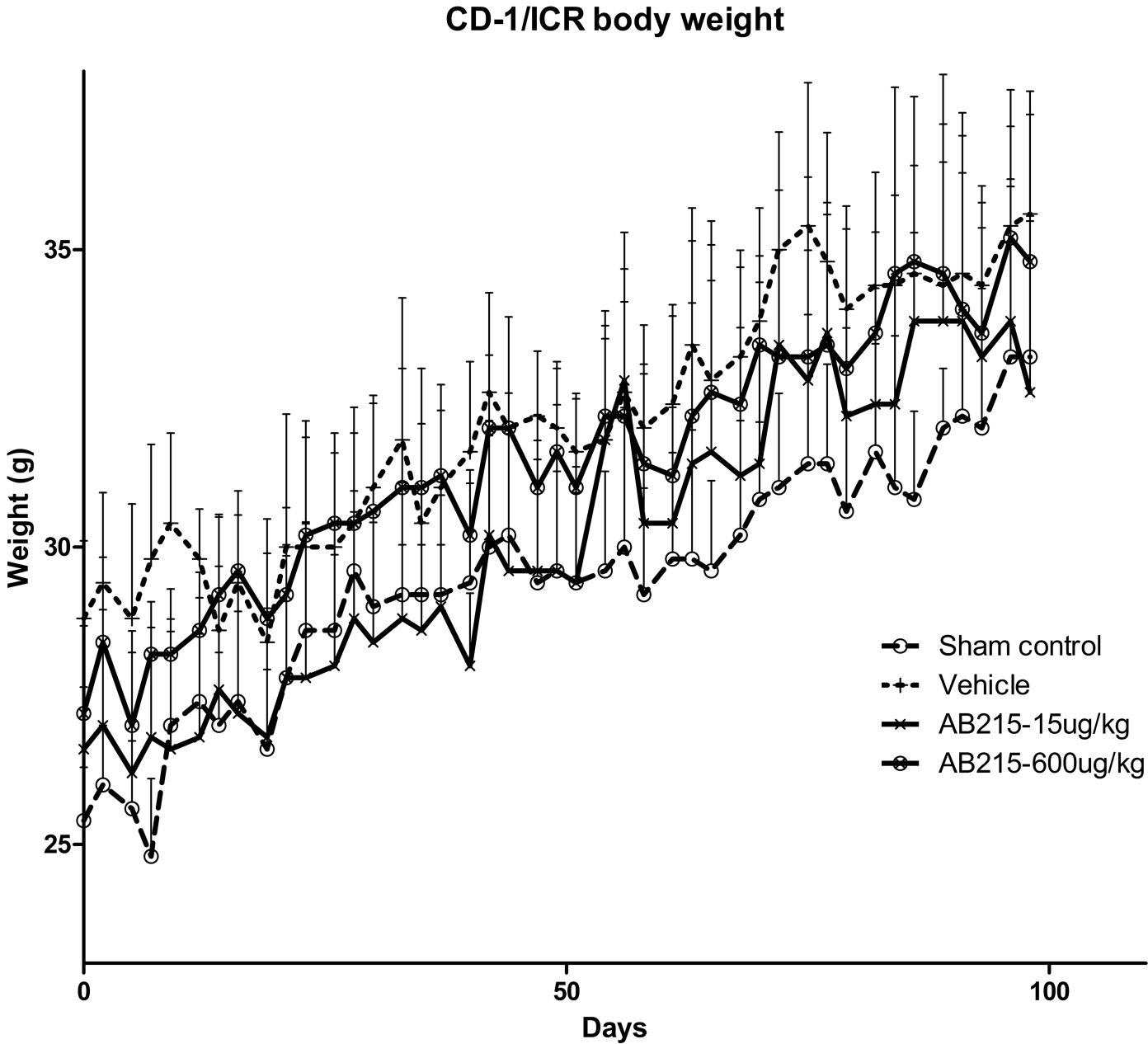

Supplement: Supplementary file 3 — Additional file 3: Figure S3: Lethality test of AB215 in CD-1/ICR mice. To verify the lethality and ectopic organ bone formation of the AB215 in the body, AB215 was injected intraperitoneally injected three times a week for 98 day at two concentrations (15 ug/kg and 600 ug/kg, n = 5). The body weight was measured three times a week and is shown as mean + SD. (PDF 11 MB) [file 12885_2014_4723_MOESM3_ESM.pdf]

# Supplemental Figure 4

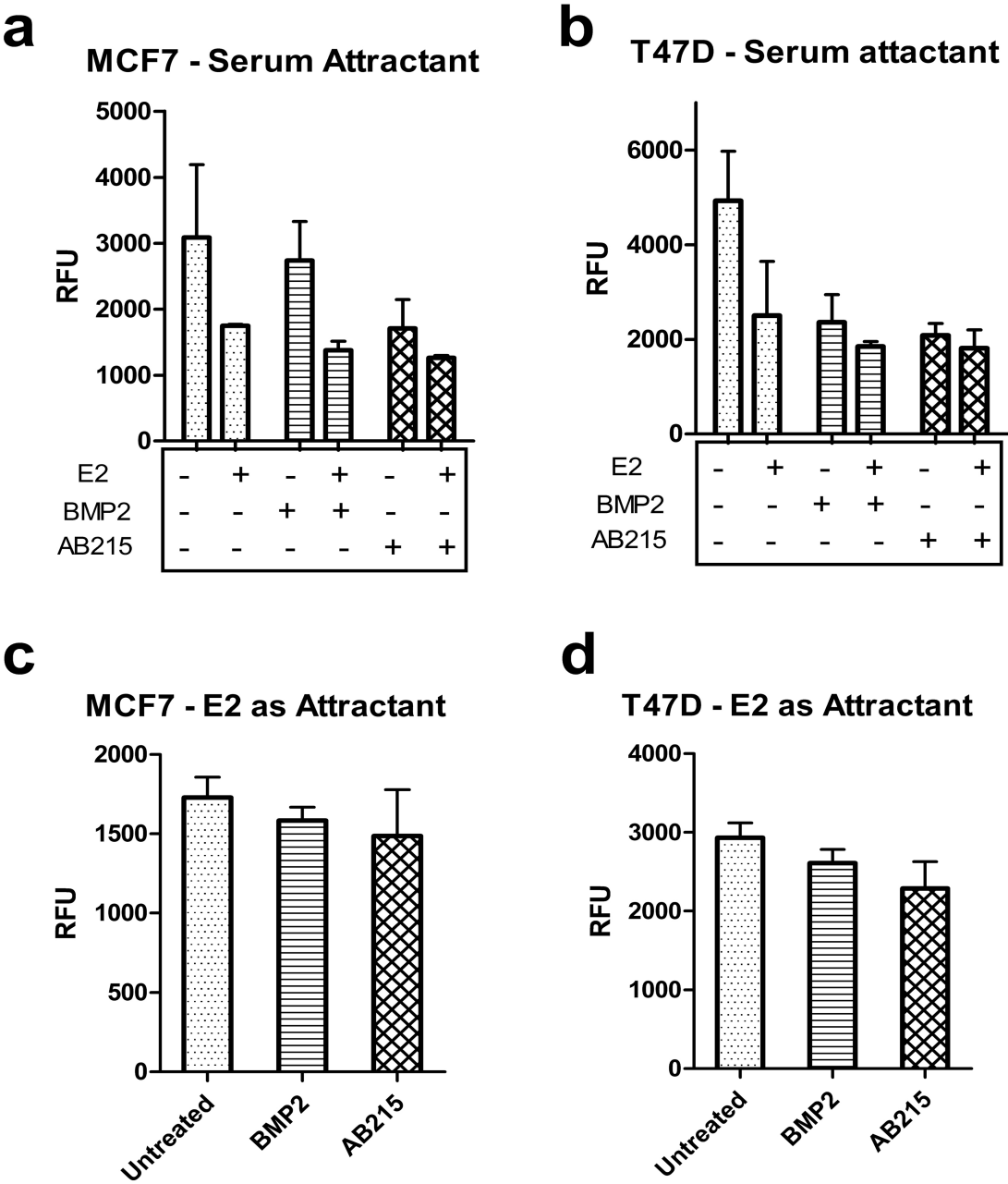

Supplement: Supplementary file 4 — Additional file 4: Figure S4: AB215 (and BMP2) in vitro basal membrane invasion test. MCF7 and T47D cells were treated as defined and analyzed for basement membrane matrix invasion ability as described in the methods section. As an attractant, 10% FBS (a and b) and 10nM E2(c and d) were used. Results are presented as means + SD. (PDF 10 MB) [file 12885_2014_4723_MOESM4_ESM.pdf]
